# How to Use Ion-Molecule Reaction Data Previously Obtained in Helium at 300 K in the New Generation of Selected Ion Flow Tube Mass Spectrometry Instruments Operating in Nitrogen at 393 K

**DOI:** 10.1021/acs.analchem.3c02173

**Published:** 2023-07-16

**Authors:** Stefan J. Swift, Patrik Španěl, Nikola Sixtová, Nicholas Demarais

**Affiliations:** † J. Heyrovsky Institute of Physical Chemistry, 3, Dolejškova 2155, Praha 8 182 00, Libeň, Czechia; ‡ Syft Technologies, 68 Saint Asaph Street, Christchurch Central City, Christchurch 8011, New Zealand

## Abstract

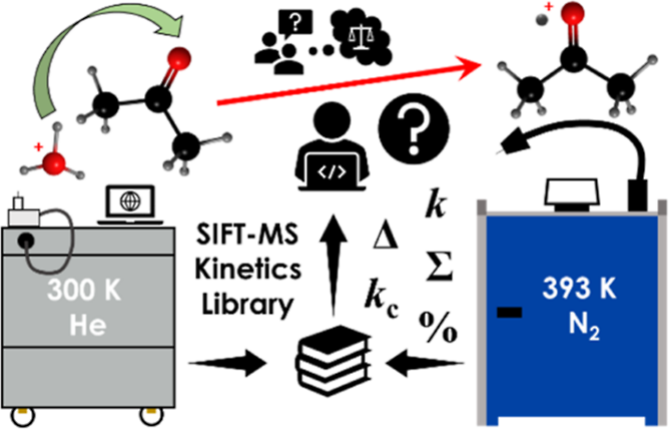

Selected ion flow tube mass spectrometry (SIFT-MS) instruments have significantly developed since this technique was introduced more than 20 years ago. Most studies of the ion–molecule reaction kinetics that are essential for accurate analyses of trace gases and vapors in air and breath were conducted in He carrier gas at 300 K, while the new SIFT-MS instruments (optimized to quantify concentrations down to parts per trillion by volume) operate with N_2_ carrier gas at 393 K. Thus, we pose the question of how to reuse the data from the extensive body of previous literature using He at room temperature in the new instruments operating with N_2_ carrier gas at elevated temperatures. Experimentally, we found the product ions to be qualitatively similar, although there were differences in the branching ratios, and some reaction rate coefficients were lower in the heated N_2_ carrier gas. The differences in the reaction kinetics may be attributed to temperature, an electric field in the current flow tubes, and the change from He to N_2_ carrier gas. These results highlight the importance of adopting an updated reaction kinetics library that accounts for the new instruments’ specific conditions. In conclusion, almost all previous rate coefficients may be used after adjustment for higher temperatures, while some product ion branching ratios need to be updated.

## Introduction

Selected ion flow tube-mass spectrometry (SIFT-MS) is a soft chemical ionization technique increasingly used globally to directly analyze volatile organic compounds (VOCs) within gaseous media.^1,2^ SIFT-MS has become a particularly popular technique in the analysis of human breath in clinical settings;^3,4^ the monitoring of the environment (i.e., atmospheric chemistry);^5−7^ headspace analysis of samples (such as foods and cosmetics);^8,9^ the analysis of contaminants within hydrogen (as a fuel);^10^ as well as airborne molecular contaminants in semiconductor manufacturing.^11^ A common theme in this diverse scope of applications of SIFT-MS is the attempt at immediate and absolute quantification of targeted VOCs.

In SIFT-MS, the reagent ions from a microwave glow discharge through a mixture of air and water are selected by a quadrupole mass filter and injected into the carrier gas (nitrogen or helium),^12^ continuously moving along the flow tube. The gaseous sample enters the flow tube via a heated capillary connected to an inlet port.^13^ Ion–molecule reactions then occur in the flow tube, converting a small fraction of the reagent ions into product ions. The resulting ion composition is analyzed by a downstream quadrupole mass spectrometer. Concentrations of gaseous analytes^14,15^ are calculated by a data system software from the measured flow tube pressure, temperature, carrier, and sample flow rates using the kinetics data (rate coefficients and product ion branching ratios) taken from a library provided by the suppliers of the SIFT-MS instruments. The inherent danger of this procedure is that using inappropriate kinetics data may lead to systematic errors in absolute quantification, compromising the accuracy of the determined concentrations. Thus, it is essential to assess all factors influencing the rate coefficients and branching ratios taken from the previous studies under realistic conditions of SIFT-MS analyses.

Historically, SIFT-MS is based on the SIFT technique for studying the kinetics of gas-phase ion–molecule reactions. SIFT-MS instruments can thus be used to determine rate coefficients and branching ratios by well-established methods.^16^ It is, however, important to note that conditions in various instruments used over the last 35 years have not always been identical. Many original studies of the ion chemistry of H_3_O^+^, NO^+^, and O_2_^+•^ reagent ions were completed on laboratory SIFT instruments with flow tubes 40 to 100 cm long, operating with He at a pressure of 0.5 Torr at a temperature of 300 K. Later, smaller instruments called Mk 1, Mk 2, *Profile 3*, Voice100, and Voice200infinity were developed and used for SIFT kinetics studies, initially in He, at pressures ranging from 0.6 to 1 Torr and temperatures between 300 and 400 K.^12,17^ Later, the N_2_ carrier gas was introduced, which is now used almost exclusively at pressures of 0.4 to 0.5 Torr and temperatures from 390 to 400 K for practical analyses using the Voice200infinity.^1,12,18^

The change in the carrier gas was the focus of our previous work using only a *Profile 3* instrument,^12,19^ where we found that N_2_ tends to fragment the injected reagent ions, but this can be mitigated by lowering the injection energy.^19^ Also, at an ambient temperature of 300 K, the relative proportion of hydrates is significantly greater in N_2_ than in He.^12^

The library of kinetics data provided with the LabSyft software (Syft Technologies, New Zealand) is, to a significant degree, based on literature values, most of which were obtained in He carrier gas (0.5 to 1 Torr, 300 K).^1^ It is the aim of the present study to assess the effects of carrier gas, flow tube temperature, and electric fields on rate coefficients and branching ratios and to indicate how historical kinetics data should be treated to achieve accurate analyses using the current instruments. Thus, we have conducted a comparison between the previous *Profile 3* results (1 Torr He at 300 K) and the present results obtained in the *Profile 3* (0.2 Torr N_2_ at 300 K) and Voice200infinity (0.46 Torr N_2_ at 390 K). As model analytes, a range of small- to medium-sized VOCs were chosen: 1-propanol, 2-propanol, 2,3-butanedione, acetaldehyde, acetic acid, acetone, ethyl acetate, ethanol, and *R*-limonene. This investigation has provided an insight into the factors affecting the kinetics data and has resulted in suggestions on how the library data should be reviewed and adjusted.

## Experimental Section

In the present study, N_2_ carrier gas was used exclusively. Voice200infinity employs positive and negative reagent ions for compound detection and quantification; however, as *Profile 3* is only able to inject positive ions (H_3_O^+^, NO^+^, and O_2_^+•^), only these three ions were included. The rate coefficients, *k*, were determined relative to the calculated H_3_O^+^ collisional rate coefficient, *k*_c_, from reductions of reagent ion signals with the addition of VOC vapor. The product ion branching ratios were determined by extrapolation to the limit of zero VOC concentration.^12^ The measurements were completed simultaneously on both instruments placed side by side and sampling from the same vessel. A blank analysis was also conducted before each set of measurements. Details are described in Section S1 of the Supporting Information.

### SIFT-MS Instrumentation

The two instruments used in this study are *Profile 3* and Voice200infinity. The key differences relevant for the present study are different flow tube pressures and temperatures as well as the arrangement of the electric fields at the end of the flow tube.

### Profile 3 Instrument

**Figure 1:**
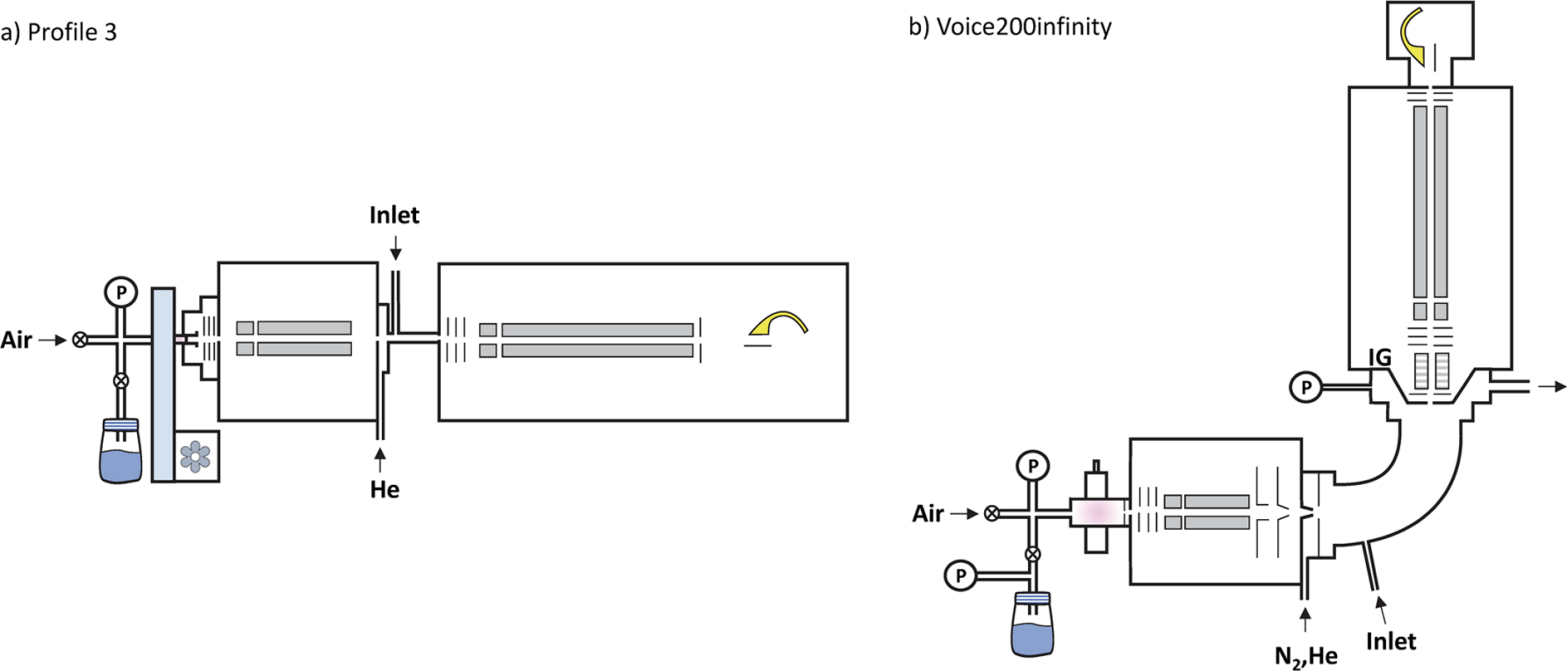
Schematic diagrams of *Profile 3* (a) and Voice200infinity (b) instruments drawn to the same scale.

*Profile 3* (Instrument Science, Crewe, UK) was used in conjunction with the *Profile 3* SIFT-MS/FA-MS data system 3.1.414.1644 software. The flow tube had a length of 5 cm, diameter of 1 cm (see Figure 1a), and the reaction time was 0.38 ms. The flow tube temperature was 300 K and the pressure was 200 mTorr. The sample inlet was realized using *ca*. 10 cm of 0.18 mm (internal diameter) PEEK capillary tubing, which gave an inlet flow rate into the instrument of 23 sccm. This inlet was heated to 323 K. Mass discrimination and differential diffusion are accounted for in ion signal analyses.^15,20^

### Voice200infinity Instrument

Voice200infinity (Syft Technologies, New Zealand) was used in conjunction with Kiosk v3.3.25. Data analysis was conducted using Labsyft v1.8.2 (Syft Technologies, New Zealand). The reaction flow tube was bent to 90° and had an axial length of 15 cm and inner diameter of 6 cm (see Figure 1b), which resulted in a reaction time of ∼5 ms. The temperature of the flow tube was set to 393 K and the flow tube pressure was 460 mTorr. The inlet was heated to 323 K and was also made up of *ca*. 10 cm of 0.18 mm (internal diameter) PEEK capillary tubing and gave an inlet flow rate of *ca*. 25 sccm. Instrument correction factors and ion guide attenuation factors were determined and applied as recommended by the manufacturer during the setup and validation of the instrument.

### Chemicals

A wide scope of chemical classes of VOCs were included in order to ascertain the differences in ion–molecule reactions between the literature data and the present data obtained from the two instruments. We therefore used 1-propanol (PENTA, 99.5%), 2-propanol (PENTA, 99.8%), 2,3-butanedione (Aldrich, 97%), acetaldehyde (Sigma-Aldrich, ≥99.5%), acetic acid (Sigma-Aldrich, ≥99.7%), acetone (PENTA, 99.5%), ethyl acetate (Sigma-Aldrich, 99.8%), ethanol (Lachner, 96%), and *R*-limonene (Sigma-Aldrich, Analytical Standard).

## Results and Discussion

The branching ratios of the primary product ions for the 27 ion–molecule reactions are given in Table 1 as reported in the literature for the previous studies carried out in He at various pressures at 300 K on different instruments, together with the present data obtained in N_2_ at 300 K from *Profile 3* and 393 K from Voice200infinity. The summary formulae for all product ions were assigned to observed *m*/*z* values, following the previous work or considering the most plausible fragmentation. Table 2 summarizes the literature data on rate coefficients together with molecular parameters involved in the calculation of *k*_c_, and Table 3 gives the new data on the rate coefficients obtained by the two instruments in the present study. These are the data resulting from this study and they can now be discussed in terms of both how they differ from the previous work and what the differences are between the two instruments. Some trends that can be identified in this substantial model set of data are relevant to the applicability of the previous compiled libraries and to future Voice200infinity analyses performed using N_2_ carrier at 393 K.

**Table 1:** Primary Product Ions of the Reactions of H_3_O^+^, NO^+^, and O_2_^+•^ with the VOC Listed^a^

	product ion	*m*/*z*			H_3_O^+^	product Ion	*m/z*			NO^+^	product Ion	*m*/*z*			O_2_^+•^
**acetaldehyde**	C_2_H_5_O^+^	45	100^c^	*100*	**99**	C_2_H_4_O·NO^+^	74	27^f^	*19*	**4**	C_2_H_4_O^+•^	44	55^c^	*7*	**3**
C_2_H_4_O 44	C_2_H_3_O^+^	43			**1**	C_2_H_3_O^+^	43	73^f^	*81*	**96**	C_2_H_3_O^+^	43	45^c^	*93*	**97**
**ethanol**	C_2_H_7_O^+^	47	100^b^	*100*	**100**	C_2_H_6_O·NO^+^	76	7^g^	*4*		C_2_H_6_O^+•^	46	<5^i^	*5*	**0**
C_2_H_6_O 46						C_2_H_5_O^+^	45	93^g^	*96*	**100**	C_2_H_5_O^+^	45	52^i^	*22*	**65**
											CH_3_O^+^	31	48^i^	*73*	**35**
**acetone**	C_3_H_7_O^+^	59	100^c^	*100*	**98**	C_3_H_6_O·NO^+^	88	100^c^	*100*	**81**	C_3_H_6_O^+•^	58	60^c^	*65*	**34**
C_3_H_6_O 58	C_2_H_3_O^+^	43			**2**	C_3_H_6_O^+•^	58			**7**	C_2_H_3_O^+^	43	40^c^	*35*	**65**
						C_2_H_3_O^+^	43			**12**	C_2_H_2_O^+•^	42			**1**
**1-propanol**	C_3_H_9_O^+^	61	10^b^	*29*	**9**	C_3_H_8_ONO^+^	90	4^g^	*5*		C_3_H_8_O^+•^	60		*10*	**3**
C_3_H_8_O 60	C_3_H_7_^+^	43	90^b^	*71*	**91**	C_3_H_7_O^+^	59	96^g^	*95*	**100**	C_3_H_7_O^+^	59		*2*	**24**
											CH_5_O_2_^+^	49		*6*	**12**
											C_3_H_6_^+•^	42	10^b^	*2*	**10**
											CH_3_O^+^	31	90^b^	*76*	**48**
**2-propanol**	C_3_H_8_OH^+^	61	20^b^	*39*	**20**	C_3_H_7_O^+^	59	100^b^	*91*	**92**	C_3_H_7_O^+^	59		*22*	**4**
C_3_H_8_O 60	C_3_H_7_^+^	43	80^b^	*61*	**80**	C_2_H_5_O^+^	45		*7*	**7**	C_2_H_5_O^+^	45	100^b^	*60*	**77**
						C_3_H_7_^+^	43		*2*	**1**	C_2_H_4_O^+•^	44		*4*	**3**
											C_3_H_7_^+^	43		*14*	**16**
**acetic acid**	CH_3_COOH_2_^+^	61	100^d^	*93*	**94**	CH_3_COOH·NO^+^	90	100^d^	*100*	**88**	CH_3_COOH^+•^	60	50^d^	*66*	**58**
CH_3_COOH 60	C_2_H_3_O^+^	43		*7*	**6**	C_2_H_3_O^+^	43			**12**	COOH^+^	45		*0*	**2**
											C_2_H_3_O^+^	43	50^d^	*34*	**40**
**2,3-butanedione**	C_4_H_7_O_2_^+^	87	100^c^	*92*	**79**	C_4_H_6_O_2_·NO^+^	116	17^h^	*18*	**4**	C_4_H_6_O_2_^+•^	86	20^c^	*33*	**5**
C_4_H_6_O_2_ 86	C_3_H_7_O^+^	59		*6*	**10**	C_4_H_6_O_2_^+•^	86	83^h^	*81*	**17**	C_2_H_3_O^+^	43	80^c^	*67*	**95**
	C_2_H_3_O^+^	43		*2*	**11**	C_2_H_3_O^+^	43		*1*	**79**					
**ethyl acetate**	C_4_H_8_O_2_H^+^	89	100^d^	*80*	**86**	C_4_H_8_O_2_·NO^+^	118	90^d^	*100*	**85**	C_4_H_8_O_2_^+•^	88		*12*	**6**
C_4_H_8_O_2_ 88	C_2_H_5_O_2_^+^	61		*17*	**10**	C_2_H_3_O^+^	43	10^d^		**7**	C_3_H_5_O_2_^+^	73		*12*	**1**
	C_2_H_3_O^+^	43		*3*	**4**	other				**8**	C_2_H_5_O_2_^+^	61	40^d^	*24*	**38**
											C_2_H_5_O^+^	45	20^d^	*24*	**19**
											C_2_H_3_O^+^	43	20^d^	*25*	**33**
											CH_3_O^+^	31	20^d^		
											other			*3*	**3**
***R*-limonene**	C_10_H_17_^+^	137	80^e^	*63*	**43**	C_10_H_16_·NO^+^	166	2^e^	*0*	**1**	C_10_H_16_^+•^	136	11^e^	*13*	**6**
C_10_H_16_O_3_ 136	C_6_H_9_^+^	81	20^e^	*17*	**34**	C_10_H_16_^+•^	136	94^e^	*93*	**68**	C_9_H_13_^+^	121	13^e^	*13*	**12**
	Other			*20*	**23**	other		4^e^	*7*	**31**	C_7_H_10_^+•^	94	11^e^	*17*	**13**
											C_7_H_9_^+^	93	26^e^	*20*	**27**
											other		39^e^	*37*	**42**

a The branching ratios are given as percentages as obtained in He at 300 K in previous studies (normal font); in *Profile 3* with N_2_ carrier gas at 300 K (italics); and in Voice200infinity with N_2_ carrier gas at 293 K (bold).

b Španěl et al., (1997).^22^

c Španěl et al., (1997).^23^

d Španěl et al., (1998).^24^

e Španěl et al., (2022).^12^

f Smith et al., (2014).^25^

g Španěl et al., (2017).^26^

h Smith et al.(2019).^27^

i Bruhová-Michalcíková & Španěl (2014).^28^

**Table 2:** The Previously Determined Theoretical Rate Constants (*k*_c_, 10^–9^ cm^3^ s^–1^) and Experimental Rate Constants (*k*, 10^–9^ cm^3^ s^–1^) for the Reactions of Each of the Nine Analyte Species Investigated in This Work with the H_3_O^+^, NO^+^, and O_2_^+•^ Reagent Ion Species^a^

compound	mass/gmol^–^^1^	IE/eV	PA/kJ mol^–^^1^	polarizability, α Å^3^ (× 10^–^^24^cm^3^)	carrier gas	H_3_O+ *k*, [*k*_c_]	NO^+^ *k*, [*k*_c_]	O_2_^+•^ *k*, [*k*_c_]	reference
1-propanol	60	10.2	787	6.7	He	2.7, [2.7]	2.3, [2.3]	2.2, [2.2]	Španěl et al^22^
2-propanol	60	10.2	793	7.6	He	2.7, [2.7]	2.4, [2.3]	2.3, [2.3]	Španěl et al^22^
2,3-butanedione	86	9.3	802	8.2	He	1.7, [1.7]	1.3, [1.4]	1.4, [1.4]	Španěl et al^23^
					He	1.7, [1.7]	1.7, [1.4]	1.6, [1.4]	Smith et al^27^
acetaldehyde	44	10.2	769	4.6	He	3.7, [3.7]	0.6, [3.2]	2.3, [3.1]	Španěl et al^23^
					He		0.93		Smith et al^25^
acetic acid	60	10.7	784	5.1	He	2.6, [2.6]	0.9, [2.2]	2.3, [2.2]	Španěl et al^24^
acetone	58	9.7	812	6.3	He	3.9, [3.9]	1.2, [3.3]	2.7, [3.3]	Španěl et al^23^
ethyl acetate	88	10.0	836	9.7	He	2.9, [2.9]	2.1, [2.4]	2.0, [2.4]	Španěl et al^24^
ethanol	46	10.5	776	5.1	He	2.7, [2.7]	1.2, [2.3]	2.3, [2.3]	Španěl et al^22^
*R*-limonene	136	8.3	836	17.6	He	2.5, [2.5]	2.2, [2.1]	2.2, [2.0]	Španěl et al^12^
*R*-limonene	136	8.3	836	17.6	N_2_	2.5, [2.5]	2.0, [2.1]	2.0, [2.0]	Španěl et al^12^

a The majority of this work was carried out using *Profile 3* with He carrier gas. The molecular parameters for each analyte are also shown, for which the data have been taken from the NIST Chemistry Webbook.^32^

**Table 3:** The Calculated Collisional Rate Constants (*k*_c_, 10^–9^ cm^3^ s^–1^) as Well as the Experimental Rate Constants (*k*, 10^–9^ cm^3^ s^–1^) for the Reactions of Each of the Nine Analyte Species Investigated in This Work with the Three Positive Reagent Ion Species, Comparing *Profile 3* (with a Flow Tube Temperature of 300 K) and Voice200infinity (with a Flow Tube Temperature of 393 K) SIFT-MS Instruments

	reagent	H_3_O^+^		NO^+^		O_2_^+•^	
	instrument	*Profile 3*	**Voice200infinity**	*Profile 3*	**Voice200infinity**	*Profile 3*	**Voice200infinity**
compound	**dipole**/debye	[*k*_c_]	[*k*_c_]	*k*, [*k*_c_]	*k*, [*k*_c_]	*k*, [*k*_c_]	*k*, [*k*_c_]
1-propanol	1.68	2.71	2.49	2.3, [2.3]	1.8, [2.1]	2.3, [ 2.3]	2.2, [2.1]
2-propanol	1.66	2.75	2.54	2.3, [2.3]	2.2, [2.2]	2.3, [2.3]	2.2, [2.1]
2,3-butanedione	0.05	1.71	1.71	1.4, [1.4]	1.4, [1.4]	1.4, [1.4]	1.4, [1.4]
acetaldehyde	2.69	3.72	3.36	0.8, [3.2]	0.5, [2.9]	3.2, [3.2]	2.9, [2.8]
acetic acid	1.74	2.64	2.42	0.8, [2.2]	0.2, [2.1]	2.5, [2.2]	2.1, [2.0]
acetone	2.88	3.92	3.54	2.0, [3.3]	0.9, [3.0]	3.3, [3.3]	3.0, [3.0]
ethyl acetate	1.78	2.89	2.68	2.4, [2.4]	1.7, [2.2]	2.4, [2.4]	2.2, [2.2]
ethanol	1.69	2.68	2.46	1.3, [2.3]	1.3, [2.1]	2.3, [2.3]	2.8, [2.1]
*R*-limonene	0.49	2.54	2.52	2.2, [2.1]	2.3, [2.1]	2.1, [2.0]	2.2, [2.0]

A detailed comparison between the results obtained by the two instruments (under their standard conditions) is made and discussed for all 27 reactions in the Supporting Information. Below, we summarize some of the trends observed in the data.

### Trends in the Observed Product Ion Branching Ratios

The product ions that were previously reported are almost always observed by both instruments in the present study. The only exception is a minor product of the O_2_^+•^ reaction with ethyl acetate (CH_3_O^+^) at *m*/*z* 31, which is completely absent in the new data. Overall, this is very good news as it indicates that using the previously determined product ions is not going to lead to missing analytes. On the other hand, additional and often minor products are observed in N_2_.

These minor products include C_2_H_3_O^+^ (*m*/*z* 43) for the H_3_O^+^ reactions with acetaldehyde, acetone, acetic acid, 2,3-butaedione, and ethyl acetate as well as C_3_H_7_O^+^ (*m*/*z* 59) for 2,3-butanedione and C_2_H_5_O_2_^+^ (*m*/*z* 61) for ethyl acetate. For the NO^+^ reactions in N_2_, C_2_H_5_O^+^ (*m*/*z* 45) and C_3_H_7_^+^ (*m*/*z* 43) were additionally observed for the reaction with 2-propanol and C_2_H_3_O^+^ (*m*/*z* 43) was observed with 2,3-butanedione. For the reactions of O_2_^+•^ with 1-propanol, 2-propanol, acetic acid, and ethyl acetate, several additional product ions appear when using N_2_ as the carrier gas (see Table 1). Also worthy of note is that some minor product ions are observed only with the Voice200infinity, while they were absent in the N_2_ carrier gas *Profile 3* spectra. This is manifested for three NO^+^ reactions (see Table 1).

The degree of fragmentation is simply indicated by the percentage of the remaining protonated molecules, NO^+^ adducts, or the radical cation products of the O_2_^+•^ charge transfer. In general, this percentage is reduced in the Voice200infinity data (bold in Table 1) in comparison with the *Profile 3* values (italics in Table 1); there are only four exceptions to this rule, all within 1 to 6%. A good example of this are the O_2_^+•^ acetone product ions where in *Profile 3*, the signal ratio of *m*/*z* 58 to *m*/*z* 43 is 2:1, while in Voice200infinity, it is reversed to 1:2.

### Effect of the Carrier Gas Type

Converting the carrier gas from He to N_2_ is seen to result in some measurable changes in ion product branching ratios with respect to the previous work. For H_3_O^+^ reactions with acetaldehyde, ethanol, and acetone, there is no change, and non-dissociative proton transfer is the only process. However, the H_3_O^+^ reactions of both isomers of propanol result in a majority C_3_H_7_^+^ product ions at *m*/*z* 43, the fraction of which is reduced in N_2_ at 300 K and increased again at 393 K. This can be explained by more efficient collisional stabilization of the nascent reaction intermediate ion.^21^

However, for 2,3-butanedione, acetic acid, and ethyl acetate (which in previous He 300 K studies resulted only in a single ion product represented by the protonated molecule), additional fragment ions (up to 21%) are observed in the present results obtained in N_2_ in both instruments. For the representative monoterpene, the ratio of the main product at *m*/*z* 137 to the dominant fragment at *m*/*z* 81 (4:1) is not changed in *Profile 3* when replacing He by N_2_. However, in Voice200infinity, at 393 K, this ratio changes close to 7:4. Note that the present results include multiple other apparent fragment product ions. In the NO^+^ reactions, somewhat surprisingly, the relative signal of the minor adduct ion product in N_2_ is smaller for acetaldehyde and ethanol in comparison with the previous He work. Again, the Voice200infinity results include small percentages of additional fragment product ions (see Table 1). The outstanding case is 2,3-butanedione, where the fragment at *m*/*z* 43, resulting from splitting the molecule into two halves, increases its relative intensity to 79%. The O_2_^+•^ products always include fragments, and the overall trend is that the degree of fragmentation decreases in N_2_ in comparison to He but increases again with increased temperature in Voice200infinity. This can be explained by the fact that the N_2_ molecule is 7 times heavier compared to He and therefore is more efficient in quenching the excited reaction intermediates. Note that the amount of O_2_^+•^ hydration is minimal, especially within the much higher temperature Voice200infinity (<0.001% O_2_^+•^ H_2_O for the Voice200infinity and <1% O_2_^+•^.H_2_O for the *Profile 3*). As a result, the influence of hydration on O_2_^+•^ is negligible.

### Effect of Temperature and Electric Fields

An extra set of electric fields are located at the end of the flow tube in Voice200infinity for efficient ion extraction that are not present in *Profile 3*. Voice200infinity can reach much greater count rates compared to the *Profile 3* instrument partly due to the presence of electric fields within the flow tube as a result of the potential difference between the flow tube wall and the sampling orifice. The presence of the extra electric fields at the end of the flow tube (as well as the increase in flow tube temperature) is a possible explanation for the observed differences in the branching ratios listed in Table 1 (now referring to the italics and bold columns). A good example of this in the H_3_O^+^ products are 1-propanol and 2-propanol, where a greater proportion of the C_3_H_7_^+^ fragment ion is observed using the Voice200infinity instrument compared to *Profile 3* in N_2_. It is possibly just a coincidence that these proportions (within Voice200infinity using N_2_) are similar to the *Profile 3* results in He. N.B. In newer models of the SIFT-MS, the carrier gas has undergone a transition from He to N_2_ due to the rising costs of He and its unsustainability.

Likewise, for NO^+^ (as mentioned before), 2,3-butanedione produces C_2_H_3_O^+^ as the major product (79%). It should be noted that this is practically absent in *Profile 3* (0 in He and 1% in N_2_). Also, the NO^+^ with acetone and acetic acid reactions in Voice200infinity leads to a substantial fraction (12%) of C_2_H_3_O^+^ at *m*/*z* 43, which was not detected in *Profile 3*. The NO^+^ reactions with acetaldehyde and ethyl acetate also show significantly higher branching ratios for the fragmentation products in Voice200infinity compared to *Profile 3* (Table 1). There is also a clear trend in the NO^+^ adduct ion percentage. It is always lower in Voice200infinity, which is explained by the lower flow tube temperature of *Profile 3* that encourages the adduct formation process. The adduct, however, is more susceptible to break-up in the presence of the high temperature and the electric field in the Voice200infinity flow tube. As a result, the process of adduct formation is more efficient in the cooler *Profile 3* flow tube compared to that in the Voice200infinity flow tube.

In general, the branching ratios of the O_2_^+•^ reaction products with all VOCs included in this study always indicate that the percentage of the molecular radical cation (listed in the first row for each VOC) is higher in *Profile 3* compared to Voice200infinity. This is particularly evident for 1-propanol, 2-propanol, 2,3-butanedione, acetone, and ethyl acetate, as demonstrated in Table 1. This may be explained by the lower temperature of the *Profile 3* flow tube (300 K) compared to the Voice200infinity flow tube (393 K) and by the presence of the electric field in the flow tube. As O_2_^+•^ is the only radical out of the three positive reagent ions, it is the highest energy positive reagent ion and commonly causes fragmentation in ion–molecule reactions. Due to the increased flow tube temperature as well as the ion optics, this fragmentation is more pronounced within the Voice200infinity instrument compared to *Profile 3*.

### Reaction Rate Coefficients and Adduct Formation

The rate coefficients, *k*, for the reactions of the positive reagent ions experimentally determined during simultaneous measurement using both SIFT-MS instruments are shown in Table 3 as calculated by the traditional method in which all three reagent ions were injected into the flow tube simultaneously and their signals depleted by at least 1 order of magnitude. A summary of the previous work that was conducted in He at 300 K over the past three decades is shown in Table 2. Note that some of the rate coefficients, *k*, obtained were found to be greater (notably for O_2_^+•^ ethanol reaction) than the calculated *k*_c_. This could be either due to experimental uncertainty or due to the process of a long-distance charge transfer explored previously.^29,30^

### Effect of the Carrier Gas Type

Comparing the previous work summarized in Table 2 (in He carrier gas) with the *Profile 3* values obtained from this work using a N_2_ carrier gas, it can be seen that for the majority of the NO^+^ reactions (with the exception of acetic acid, which stays the same), the rate coefficient values obtained in the N_2_ carrier gas at 300 K are higher compared to previous work using He. For the O_2_^+•^ reactions, this is observed to a small degree for all rate coefficient comparisons. The rate coefficients are generally higher within N_2_ carrier gas due to the increased susceptibility of adduct formation in both cases despite the adduct branching ratios generally decreasing in N_2_ carrier gas compared to He (see previous sections).

### Effect of Temperature

It is clear that the values of the collisional rate coefficient, *k*_c_, for all compounds are lower in the Voice200infinity instrument, which is as a direct result of the calculation used by Su and Chesnivich^31^ in which the temperature input is increased from 299 K (300 K) to 393 K (393 K). As a direct result, the *k*_c_ values for the Voice200infinity instrument are lower by around 1–10% (as seen in Table 3). The reason for this is that at higher temperatures, the rotations of the molecules are more energetic and the effect of increase of the average dipole moment of a rotating polar molecule in the vicinity of charged ions is less pronounced.^21^

**Figure 2:**
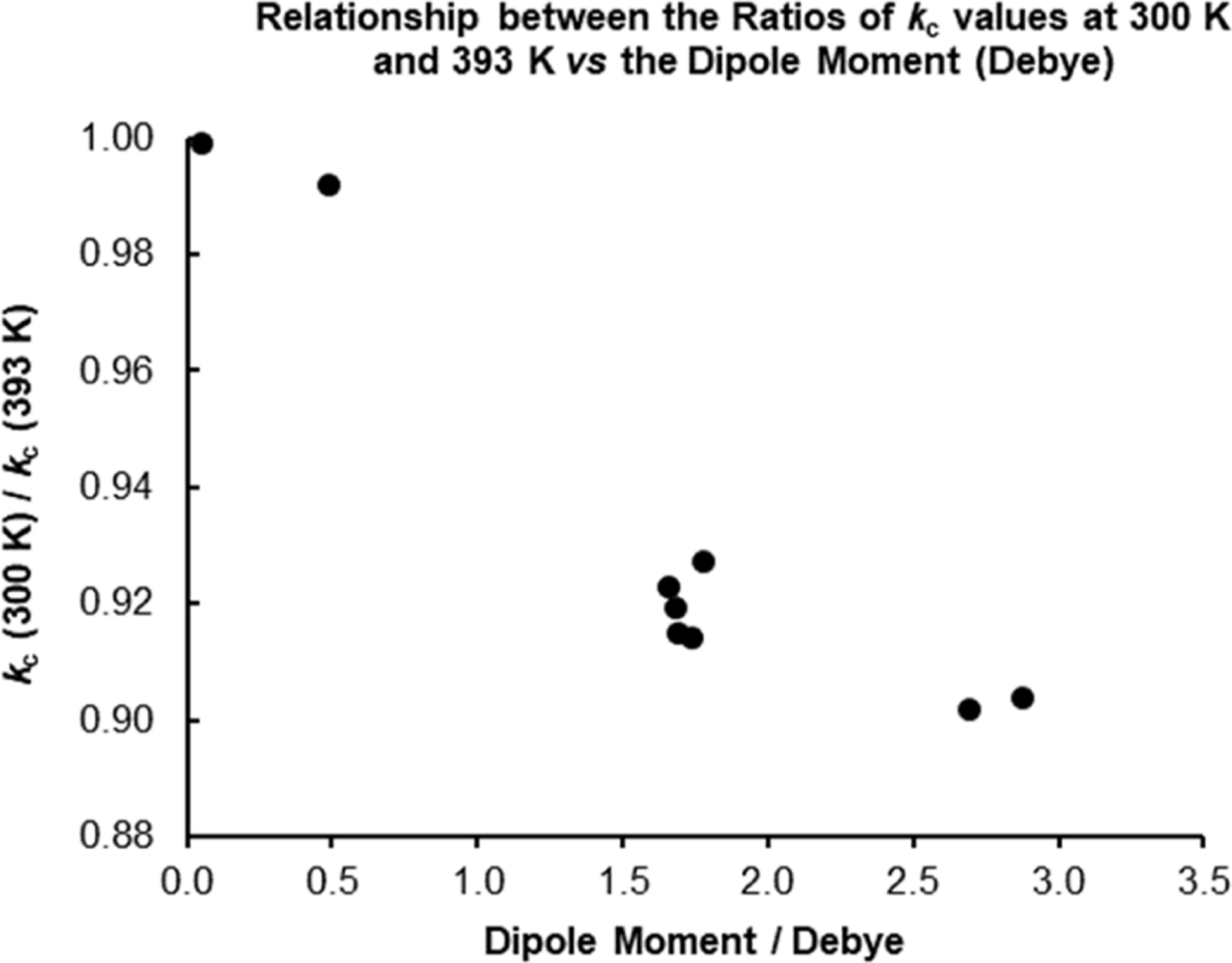
Correlation of the Dipole Moment (Debye) of molecules against the ratio of the theoretical collisional correlation coefficient for reactions of the analyte species with H_3_O^+^ at flow tube temperatures of 300/393 K.

Therefore, is not surprising that the ratio of the collisional rate coefficient at 300 K to the collisional rate coefficient at 393 K correlates with the dipole moment for each molecule (as depicted in Figure 2).

As the determined NO^+^ and O_2_^+•^ rate coefficients are proportional to the H_3_O^+^*k*_c_, the empirical rate coefficients for the NO^+^ and O_2_^+•^ reagent ions also show that all the higher temperature Voice200infinity rate coefficients are lower compared to those of *Profile 3*. The only exception is the O_2_^+•^ reaction with ethanol, for which *k* is larger in Voice200infinity, and *R*-limonene, which demonstrates the same rate coefficient for O_2_^+•^ and NO^+^ reactions in both instruments (for both reagents).

## Conclusions

A comprehensive analysis of the differences in the ion–molecule reaction kinetics observed in two SIFT-MS instrument models in N_2_ carrier gas at different temperatures has been conducted, and the results were compared to previous studies carried out in He at 300 K. The product ions were mostly similar. In some instances, a greater number of product ion species were found in the Voice200infinity instrument. The branching ratios for the reagent ions were different between instruments, especially for the O_2_^+•^ reagent ion, which induces extensive fragmentation across the VOCs compared to H_3_O^+^ or NO^+^. The systematic differences were also observed as the result of a change from He to N_2_ carrier gas. For example, the H_3_O^+^ reactions with 2,3-butanedione, acetic acid, and ethyl acetate lead to fragment ions (up to 21%) in N_2_ in both instruments that were not reported in the previous He work.

The objective of this study of 27 reaction systems (determining the branching ratios and rate coefficients) was to ascertain how the data currently included in the library on the basis of the previous He 300 K work can be used for quantification of gaseous analytes. Can they be directly transferred from the already established and comprehensive library, predominantly produced by the *Profile 3* instrument using He as the carrier gas at 300 K?

We have found that the greatest differences between the instruments are due to different carrier gas temperatures and the presence of electric fields that reduce adduct formation (directly affecting the rates of association of NO^+^ reactions). Also, the fragmentation observed in the Voice200infinity, which operated with the default factory settings, is somewhat greater. This could be caused by the differences in temperatures of the two flow tubes but possibly also by fragmentation occurring just behind the downstream sampling orifice in the ion guide region of Voice200infinity.

It was noted that an increased temperature inherently causes the theoretical *k*_c_ values to be reduced for polar molecules, which directly influences the reaction rate coefficients.

In summary, the following generalized recommendation can be formulated:

(1) The rate coefficients for the polar analytes should be recalculated using the Su and Chesnavich^31^ parametrization for the temperature of 390 K. Non-polar analytes are not affected.
(2) The rate coefficients for the NO^+^ association reactions need to be experimentally determined under the conditions of instruments operating in N_2_ at elevated temperatures as they will be generally unpredictably different from the 300 K He values.
(3) For the case of analyses that rely on a specific value of branching ratio, this value needs to be determined under the conditions of the actual instrument in use.
(4) In all other cases, including all nonpolar compounds, the previous data may be used with confidence.

The data published on the kinetics of gas phase reactions of the H_3_O^+^, NO^+^, and O_2_^+•^ ions with volatile analyte molecules cover thousands of reactions measured in He at ambient laboratory temperature (nominally 300 K). It would therefore be a shame if all of this work would have to be repeated in heated N_2_ carrier gas. Fortunately, based on the understanding of the trends discussed in this article, much of these data can be reused after considering the four points above.

## Supporting Information Available

Detailed discussion of each of the 27 individual reactions; chemical equations including the *m*/*z* values of the product ions; and their branching ratios obtained on both *Profile 3* and Voice200 instruments (PDF)
