# Supplementary material for: How to Use Ion-Molecule Reaction Data Previously Obtained in Helium at 300 K in the New Generation of Selected Ion Flow Tube Mass Spectrometry Instruments Operating in Nitrogen at 393 K
Source: Anal Chem. 2023 Jul 16;95(29):11157–63. doi: 10.1021/acs.analchem.3c02173 (PMC10372871; doi:10.1021/acs.analchem.3c02173)
Supplement: Supplementary file 1 — ac3c02173_si_001.pdf [file ac3c02173_si_001.pdf]

Supporting Information: How to use previous ion-molecule reaction data obtained in helium at 300 K in the new generation of selected ion flow tube mass spectrometry instruments operating in nitrogen at 393 K

Stefan J Swift[1], Patrik Španěl[1], Nikola Sixtová[1] and Nicholas Demarais[2]*

[1]J. Heyrovsky Institute of Physical Chemistry, 3, Dolejškova 2155, Libeň, 182 00 Praha 8, Czechia
[2]Syft Technologies, 68 Saint Asaph Street, Christchurch Central City, Christchurch 8011, New Zealand

Contents

1 CALCULATION OF BRANCHING RATIOS AND KINETIC RATE COEFFICIENTS S2

1.1 Product Ion Identification and Branching Ratios S2

1.2 Reaction Rate Coefficients S3

2 REACTIONS S3

2.1 H_3O^+ Reactions S3
2.1.1 1-Propanol S3
2.1.2 2-Propanol S4
2.1.3 2,3-Butanedione S4
2.1.4 Acetaldehyde S4
2.1.5 Acetic Acid S5
2.1.6 Acetone S5
2.1.7 Ethyl Acetate S5
2.1.8 Ethanol S6

2.2 NO^+ Reactions S6
2.2.1 1-Propanol S6
2.2.2 2-Propanol S7
2.2.3 2,3-Butanedione S7
2.2.4 Acetaldehyde S8
2.2.5 Acetic Acid S8
2.2.6 Acetone S8
2.2.7 Ethyl acetate S9
2.2.8 Ethanol S9

2.3 O_2^+ Reactions S10
2.3.1 1-Propanol S10
2.3.2 2-Propanol S11
2.3.3 2,3-Butanedione S11
2.3.4 Acetaldehyde S11
2.3.5 Acetic Acid S12
2.3.6 Acetone S12
2.3.7 Ethyl acetate S13
2.3.8 Ethanol S13

2.4 *R*-Limonene Reactions S14

1 Calculation of Branching Ratios and kinetic rate coefficients

1.1 Product Ion Identification and Branching Ratios

A clean glass vessel (250 ml) was baked over night to rid it of any volatiles. Three holes were drilled into its cap for the two inlets of the instruments and for the injection of headspace or zero-air at 120 sccm. The air within the glass vessel was displaced by zero-air before measurements began. Full *m/z* scans from *m/z* 10 to 250 for H_3O^+, NO^+ and $O_2^{+\bullet}$ reagent ions were conducted prior to any injection of headspace in order to verify that the glassware was indeed scrupulously clean. For the less volatile compounds, liquid analyte was injected into the glass vessel (250 ml) and the vessel was heated using a water bath (of boiling water). A schematic of this instrumental set-up is shown in Fig. S1.

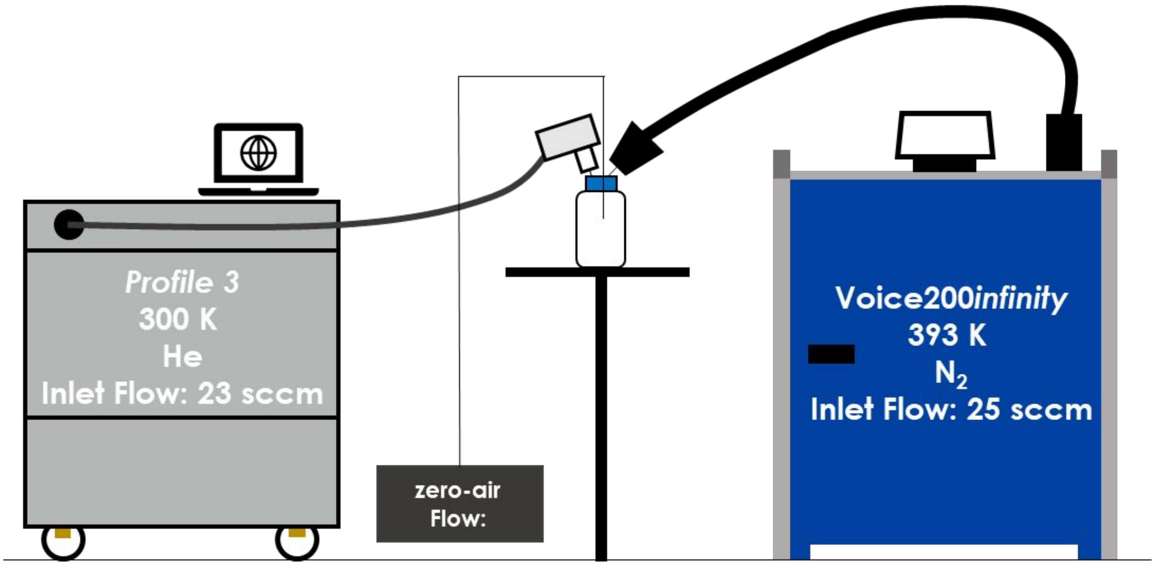

Fig. S1. Schematic of the experimental set-up of the co-sampling of the *Profile 3* and the Voce200infinity instrument during the analysis of branching ratios and kinetic reaction rate coefficients during this investigation. The sampling time for each experiment was *ca.* 10 minutes for reaction rate analyses and *ca.* 15 minutes for reaction branching ratios analyses.

On successful completion, *ca.* 2 ml of headspace from the stock bottle (of the chemicals listed in section 2.2) was injected into the glass vessel. The zero-air flow (6 sccm) was then replaced and allowed for the gradual change in concentration of the specific species to occur. As the concentration of the vapour lowered, a series of repetitive quick (30s) full scans were taken in the cycle of H_3O^+, NO^+ and $O_2^{+\bullet}$ over the course of the experiments from *m/z* 10 – *m/z* 250 (step size of 1 *m/z* unit) by both instruments simultaneously.

By compiling the mass spectra for individual reagent ions and by assessing the changes in the ion signal intensity with concentration, product ions were identified for each of the reagent ions and each of the tested chemical species. These ions were then placed into the selected-ion-monitoring (SIM) methods, used for the accurate calculation of the branching ratios.

Accurate changes of the product ion signals were recorded for all combinations of reagent ions and VOCs over a changing concentration of vapour within the glass vessel using the same approach as for the full scans, although with a larger zero-air flow rate of 120 sccm. By plotting the count rates of observed ions against a known product ion signal, a linear trend indicates a primary product and a quadratic trend indicates the presence of a secondary product. This is demonstrated for $O_2^{+\bullet}$ + 2-propanol in Fig. S2a, where *m/z* 45 is a known product ion signal ($C_2H_5O^+$), *m/z* 59 ($C_3H_7O^+$) is identified as a primary product, and *m/z* 87 (possibly $C_5H_{11}O^+$) is identified as a secondary product.

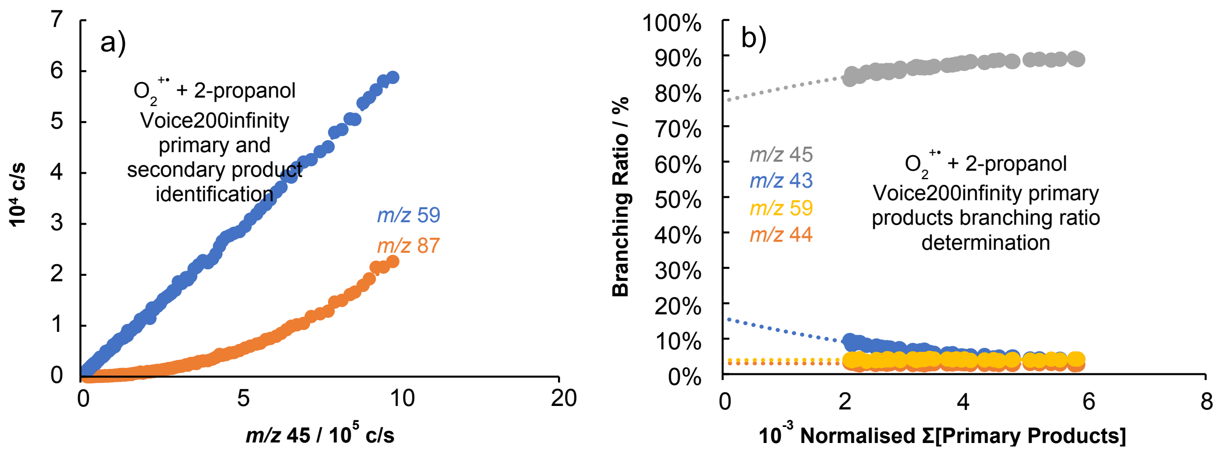

Fig. S2. a) Identifying linear and secondary correlations between the main known product ion $C_2H_5O^+$ (*m/z* 45) with a primary product (*m/z* 59) for the reaction of $O_2^{+\bullet}$ with 2-propanol, demonstrating a linear trend with *m/z* 45; as well as a secondary product (*m/z* 87), demonstrating a quadratic correlation with *m/z* 45. b) Demonstration of calculating the primary product branching ratios of the reaction of $O_2^{+\bullet}$ with 2-propanol, using the Voice200infinity. The branching ratios are taken from the extrapolation to $x = 0$ (where x is the total concentration of product ions).

The percentages of the product ion signals were plotted as functions of the VOC concentrations and the shape of the plot was assessed. The plots corresponding to the primary products were extrapolated to 0 concentration using a quadratic relationship. The intercept was taken to be the primary branching ratio (Fig. S2b). Quadratic plots with near zero intercepts were excluded, as were impurity related ions. The same analysis was carried out for the data obtained by the *Profile 3* at 300 K and for those obtained by the Voice200infinity at 393 K.

1.2 Reaction Rate Coefficients

The rate coefficients, k, were determined relative to the H_3O^+ proton transfer reaction by the well-established procedure involving simultaneous injection of all three reagent ions and observation of their decay with changing concentration of VOC vapours in the sampled air. Thus, a high concentration of VOC headspace was injected into the 250 ml blank glass vessel; in some instances (such as with *R*-limonene), a small amount of liquid needed to be injected into the heated glass vessel. The concentration of VOC within the glass vessel needed to be high enough to deplete the H_3O^+ reagent ion by at least one order of magnitude. A flow of zero-air was set to pass through the glass vessel at 70 sccm while the two instrument inlets were transporting the head space into the flow tube.

The instruments were set as to allow all reagent ions to pass through to the flow tube of the *Profile 3* as well as very quick pulses of all three reagent ions in a sequence through the Voice200infinity. During these experiments, the methods were set to record multiple-ion-monitoring (MIM) scans across the three positive reagent ions (and their hydrates). This allowed for the accurate and simultaneous measurement of the decay and re-generation of the positive reagent ions. By comparing the change in concentration of the NO^+ and $O_2^{+\bullet}$ reagent ions (and their hydrates) to that of H_3O^+ over the first order of magnitude of H_3O^+ decay (in *c/s*), the relative rates of the H_3O^+, NO^+ and $O_2^{+\bullet}$ reactions with the VOCs were established for both instruments.

Note that the proton transfer from H_3O^+ to a VOC molecule is assumed to take place at the collisional rate (k_c) for proton transfer reactions which occur at a $\Delta H < -40$ kJ mol^{-1}.[21] This can be calculated theoretically using the method described by Su and Chesnivich.[22] The relative rates of NO^+ and $O_2^{+\bullet}$ (to H_3O^+) were then multiplied by the H_3O^+ collisional rate constant (k_c) in order to obtain the experimental rate coefficient of a reaction.

2 Reactions

Table 1 and Table 2 in the main manuscript show the branching ratios and reaction rate kinetics (respectively) for all 27 reactions which have been investigated in this study. The main body of the text summarises our findings and the general reactions rules which have been indicated by the observed kinetic parameters. In this supplementary, we discuss in detail each individual reaction. In the equations below, the branching ratios have been written in square brackets, showing the *Profile 3* (italics) and Voice200 (bold) values.

2.1 H_3O^+ Reactions

For the H_3O^+ reactions, both instruments showed very similar products and branching ratios for acetaldehyde, acetic acid, acetone, ethyl acetate, and ethanol; although showed very different product branching ratios for 1-propanol, 2-propanol, 2,3-butanedione, *R*-limonene.

2.1.1 1-Propanol

For 1-propanol, both instruments produced both the charge transfer ion ($C_3H_8OH^+$, Eq. 1a) as well as the fragment ion ($C_3H_7^+$, Eq. 1b). For this reaction, a significantly higher proportion of the fragment ion was detected in the Voice200(*Infinity*) compared to the *Profile 3*. Although both instruments used N_2 as the carrier gas, the higher temperature exhibited by the Voice200 (393 K), compared to the *Profile 3* (299 K) most likely induced bond fission within the molecule causing fragmentation; over the proton transfer pathway.

$H_3O^+ + C_3H_8O \rightarrow C_3H_8OH^+ + H_2O$ (*m/z* 61) [*29*, **9**] (Eq. 1a)

$\rightarrow C_3H_7^+ + H_2O + H_2$ (*m/z* 43) [*71*, **91**] (Eq. 1b)

The original work for which the reported kinetic library is taken from for 1-propanol was conducted using a *Profile 3* SIFT-MS instrument in a He carrier gas[23]. The authors of this work reported the same two product ions, as well as branching ratios which were very similar to the Voice200(*Infinity*) instrument; which were $C_3H_7^+$ (90 %) and $C_3H_8OH^+$ (10 %). Therefore, the previously determined branching ratios reported from Španěl et al., (1997)[23] are suitable for use within the standard operating conditions of the Voice200(*Infinity*) instrument.

2.1.2 2-Propanol

Similar to 1-propanol, 2-propanol exhibits a higher branching ratio for the fragment ion within the Voice200(*Infinity*), compared to the *Profile 3*. Analogous to 1-propanol, this is most likely down to the higher temperature of the flow tube within the Voice200(*Infinity*).

$H_3O^+ + C_3H_8O \rightarrow C_3H_8OH^+ + H_2O$ (*m/z* 61) [*39*, **20**] (Eq. 2a)

$\rightarrow C_3H_7^+ + 2H_2O$ (*m/z* 43) [*61*, **80**] (Eq. 2b)

Correspondingly to 1-propanol, the branching ratios of the previous study[23] using the *Profile 3* with He also agreed with the branching ratios determined from the Voice200(*Infinity*) using N_2 in the flow tube, for which authors also reported 20 % for the proton transfer product as well as 80% for the $C_3H_7^+$ fragment. Therefore, in the case of both 1-propanol and 2-propanol, regarding the reactions with H_3O^+, the branching ratio values may be directly used in the Voice200(*Infinity*) library.

2.1.3 2,3-Butanedione

Previous work using the *Profile 3* in He showed only the production of a single product ion at *m/z* 81, being the proton transfer product (Eq. 3a). In N_2 however, an extra two minor ions were detected (Eq. 3b and 3c; 3d). There were two separate possibilities of the *m/z* 59 product which were $C_2H_3O_2^+$ (Eq.

3b) and $C_3H_6OH^+$ (Eq. 3c). ^{13}C analysis also detected the presence of 3 carbon atoms and therefore $C_3H_6OH^+$ is the most likely product here.

$$H_3O^+ + C_4H_6O_2 \rightarrow C_4H_6O_2H^+ + H_2O\ (m/z\ 87)\ [\mathit{92}, \mathbf{79}]\ \text{(Eq. 3a)}$$

$$\rightarrow C_3H_6OH^+ + CH_2O_2\ (m/z\ 59)\ [\mathit{6}, \mathbf{10}]\ \text{(Eq. 3c)}$$

$$\rightarrow C_2H_3O^+ + C_2H_3OH + H_2O\ (m/z\ 43)\ [\mathit{2}, \mathbf{11}]\ \text{(Eq. 3d)}$$

Based on the previous work by Španěl et al., (1997)[21], the proton transfer reaction was expected to be 100 %, although this previous work used He as the carrier gas within the *Profile 3*. The heavier N_2 carrier gas used in this work may be inducing fragmentation to also produce the $C_3H_6OH^+$ (Eq. 3c) and $C_2H_3O^+$ (Eq. 3d) ions. Furthermore, these minor product ions were found to increase on the injection of 2,3-butanedione headspace to the glass vessel which confirmed their reality as product ions. A peak at *m/z* 61 was also detected in this work (in both instruments), although this is most likely the hydrate of $C_2H_3O^+$ (*m/z* 47). Therefore, the branching ratios of the H_3O^+ reaction with 2,3-butanedione should be updated in the kinetics library when using the Voice200(*Infinity*) with N_2 as the carrier gas.

2.1.4 Acetaldehyde

In both instruments, the production of the proton transfer product, $C_2H_4OH^+$, was the most dominant product ion produced. This is in accordance with the previous work using the *Profile 3* with a He carrier gas.

$$H_3O^+ + C_2H_4O \rightarrow C_2H_4OH^+ + H_2O\ (m/z\ 45)\ [\mathit{100}, \mathbf{99}]\ \text{(Eq. 4a)}$$

$$\rightarrow C_2H_3O^+ + H_2 + H_2O\ (m/z\ 43)\ [\mathit{0}, \mathbf{1}]\ \text{(Eq. 4b)}$$

Therefore, with regards to the re-use of the branching ratio values, no alteration needs to be conducted to the kinetic library, although the rate of reaction (table 1) calculated at the higher flow tube temperature of the Voice200(*Infinity*) instrument does not agree with the previous study (table 2) and should therefore be updated.

2.1.5 Acetic Acid

The *Profile 3* and Voice200(*Infinity*) were in very close agreement regarding the measured branching ratios of the major proton transfer product ion (Eq. 5a). There was however also an additional minor ion produced in this reaction at *m/z* 43 (Eq. 5b). In comparison to previous studies, Španěl et al., (1997)[24] reported only one product ion ($CH_3COOH_2^+$, 100%) when using a He carrier in the *Profile 3*.

$$H_3O^+ + CH_3COOH \rightarrow CH_3COOH_2^+ + H_2O\ (m/z\ 61)\ [\mathit{93}, \mathbf{94}]\ \text{(Eq. 5a)}$$

$$\rightarrow C_2H_3O^+ + H_4O_3\ (m/z\ 43)\ [\mathit{7}, \mathbf{6}]\ \text{(Eq. 5b)}$$

It was seen that *m/z* 43 increased in this study, on the addition of acetic acid headspace to the glass vessel. $C_2H_3O^+$ (a common fragment product from many organic compounds) was therefore determined to be a real minor product ion for the reaction of H_3O^+ with acetic acid in a nitrogen carrier gas. Directly comparing both instruments in this study (using N_2), very good agreement is seen as demonstrated in Eq. 5a and Eq. 5b.

2.1.6 Acetone

Analogues to acetaldehyde, both instruments gave 100% branching ratio for the proton transfer product on the reaction between H_3O^+ and acetone (Eq. 6a and Eq. 6b). This agrees with Španěl et al., (1997)[21] who used He and a *Profile 3* instrument.

$$H_3O^+ + C_3H_6O \rightarrow C_3H_6OH^+ \ (m/z\ 59)\ [\mathit{100}, \mathbf{98}]\ \text{(Eq. 6a)}$$

$$\rightarrow C_2H_3O^+ + CH_4 + H_2O \ (m/z\ 43)\ [\mathit{0}, \mathbf{2}]\ \text{(Eq. 6b)}$$

A very minor response was also detected by the Voice200(*Infinity*) instrument at *m/z* 43 which was initially suspected to be a product ion. It was however discovered that the H_3O^+ setting on Voice200(*Infinity*) instrument was allowing some of the O_2^+ parasite ions through (at 4% the intensity of the main H_3O^+ ion peak). Therefore, the product ion at *m/z* 43 was most likely CH_3O^+ as a result of the reaction between acetone and O_2^+.

2.1.7 Ethyl Acetate

Ethyl acetate produced three product ions on the reaction with H_3O^+, which were $C_4H_8O_2H^+$ (Eq. 7a), $C_2H_5O_2^+$ (Eq. 7b) and $C_2H_3O^+$ (Eq. 7c). This is different to the previous work[24] conducted using the *Profile 3* and a He carrier gas, for which authors only reported the presence of the proton transfer product ion ($C_4H_8O_2H^+$). In this work, we also however found the presence of a substantial branching fraction of $C_2H_4O_2^+$ (*m/z* 61).

$$H_3O^+ + C_4H_8O_2 \rightarrow C_4H_8O_2H^+ + H_2O \ (m/z\ 89)\ [\mathit{80}, \mathbf{86}]\ \text{(Eq. 7a)}$$

$$\rightarrow C_2H_5O_2^+ + C_2H_4 + H_2O \ (m/z\ 61)\ [\mathit{17}, \mathbf{10}]\ \text{(Eq. 7b)}$$

$$\rightarrow C_2H_3O^+ + 2CH_4 + O_2 \ (m/z\ 43)\ [\mathit{3}, \mathbf{4}]\ \text{(Eq. 7c)}$$

Although the response of the *m/z* 61 peak correlated linearly with *m/z* 89 (the major ion), it was initially uncertain as to whether the *m/z* 61 signal was really a product ion from the H_3O^+ and ethyl acetate reaction, or not. This is because ethyl acetate is known to be able to hydrolyse in water to produce acetic acid and ethanol; for which the acetic acid (RMM 60 $gmol^{-1}$) could react with the H_3O^+ to produce the protonated acetic acid product (at *m/z* 61). Further inspection however found that the response of the *m/z* 61 signal increased on the injection of ethyl acetate headspace into the glass vessel; and no *m/z* 61 was found in the blank.

A very minor contribution of $C_2H_3O^+$ (*m/z* 43) was also detected in both instruments using N_2 as the carrier gas, in this work. This further evidences our argument that the use of N_2 as opposed to He may increase the amount fragmentation occurring during ion-molecule reactions involving H_3O^+.

2.1.8 Ethanol

The reaction of H_3O^+ with ethanol produced only the single proton transfer product ion, $C_2H_6OH^+$, which is in accordance with the previous work of Španěl et al., (1997)[23] who used the *Profile 3* with an He carrier gas.

$$H_3O^+ + C_2H_6O \rightarrow C_2H_6OH^+ + H_2O \ (m/z\ 47)\ [\mathit{100}, \mathbf{100}]\ \text{(Eq. 8)}$$

The kinetics of this reaction still however need to be updated in the kinetics library, due to the changing temperature of the flow tube inherently changing the theoretical collisional rate constant of H_3O^+ with ethanol (Tables 1 and 2), using the Su and Chesnivich., (1982)[36] method.

2.2 NO^+ Reactions

For the NO^+ reactions with the listed analytes, 1-propanol, 2-propanol and ethanol show similar branching ratios between the two instruments, whereas the other species exhibit more varying branching ratios between the product ions. NO^+ reactions specifically are prone to adduct formation and therefore it was suspected that the difference in temperatures and pressures of the flow tube between the two instruments would affect the branching ratios significantly here.

2.2.1 1-Propanol

For 1-propanol, the major product ion was the proton transfer product ($C_3H_7O^+$, Eq. 9a), although a small fraction of adduct ($C_3H_7ONO^+$) was also formed in the *Profile 3*, at 5 %.

$$NO^+ + C_3H_8O \rightarrow C_3H_7O^+ + HNO \ (m/z\ 59)\ [\mathit{95}, \mathbf{100}]\ \text{(Eq. 9a)}$$

$$\rightarrow C_3H_8O.NO^+ \ (m/z\ 90)\ [\mathit{5}, \mathbf{0}]\ \text{(Eq. 9b)}$$

The 100 % formation of the proton transfer product in the Voice200 is due to the much higher temperature of the flow tube which avoids the dative bonding of the NO^+ with the analyte molecule to form the adduct; where the higher temperatures preferentially causes bond fission and H-abstraction from the analyte to form $C_3H_7O^+$ (Eq. 9b). The lower temperature of the *Profile 3* allows for a small amount of the adduct to form ($C_3H_8ONO^+$).

In the previous work by Španěl et al., (1997)[23], the authors reported a 100% branching ratio of the H-abstraction product ($C_3H_7O^+$, Eq. 9a) which agrees with the Voice200(*Infinity*) branching ratio, under the experimental conditions discussed in our publication. It is however worthy of note that by using an N_2 carrier gas instead of He (within the *Profile 3*), this induces the formation of a minor amount of the adduct ion ($C_3H_7ONO^+$, Eq. 9b). This may be explained by the reduced speed of gaseous N_2 carrier gas molecules (under the same momentum although higher mass compared to He), which allows for softer collisions and the production of the adduct ions.

2.2.2 2-Propanol

The reaction of 2-propanol with NO^+ formed almost identical branching ratios between the *Profile 3* and Voice200(*Infinity*), when using N_2 as the carrier gas (Eq. 10a - 10c). Unlike 1-propanol, the adduct is not formed within either instrument which may be down to the secondary structure of 2-propanol causing sterical hindrance for the NO^+ to attach effectively to the alcohol structure.

$$NO^+ + C_3H_8O \rightarrow C_3H_7O^+ + HNO \ (m/z\ 59)\ [\mathit{91}, \mathbf{92}]\ \text{(Eq. 10a)}$$

$$\rightarrow C_2H_5O^+ + CH_3NO \ (m/z\ 45)\ [\mathit{7}, \mathbf{7}]\ \text{(Eq. 10b)}$$

$$\rightarrow C_3H_7^+ + HNO_2 \ (m/z\ 43)\ [\mathit{2}, \mathbf{1}]\ \text{(Eq. 10c)}$$

The previous work by Španěl et al., (1997)[23] reported a 100% production of the H-abstraction pathway (Eq. 10a), when He was used as the carrier gas. Eq. 10a – 10c infer that replacing the He carrier gas with N_2 induces some fragmentation within the *Profile 3*, for this reaction.

2.2.3 2,3-Butanedione

2,3-butanedione had one of the most radically different sets of branching ratios when comparing the two instruments. Both instruments detected the charge transfer product ($C_4H_6O_2^+$, Eq. 11a), which was the major species in the *Profile 3*; the adduct formation product ($C_4H_6O_2.NO^+$, Eq. 11b); as well as the fragment ion ($C_2H_3O^+$, Eq. 11c) which was the major species within the Voice200(*Infinity*).

$$NO^+ + C_4H_6O_2 \rightarrow C_4H_6O_2^{+\bullet} + NO^\bullet$$ (*m/z* 86) [*81*, **17**] (Eq. 11a)

$$\rightarrow C_4H_6O_2.NO^+$$ (*m/z* 116) [*18*, **4**] (Eq. 11b)

$$\rightarrow C_2H_3O^+ + C_2H_3O^\bullet + NO^\bullet$$ (*m/z* 43) [*1*, **79**] (Eq. 11c)

Initially, it was uncertain as to whether the signal at *m/z* 43 was also a product or not, although at closer inspection of the SIM time series of the experiment, it was seen that the *m/z* 43 signal increased significantly in the Voice200(*Infinity*) instrument, when the headspace of 2,3-butanedione was injected into the glass vessel.

A very large difference is seen between the branching ratios of these difference species. The fragment ion ($C_2H_3O^+$) in Eq. 11c especially, is considerably larger within the Voice200(*Infinity*) which is most likely due to the higher temperature of the flow tube encouraging bond fission, to cause the fragmentation. The temperature also explains why the adduct ion formation branching ratio (eq. 11b) is over four times lower in the Voice200(*Infinity*) compared to the *Profile 3*. A much larger fraction of the proton transfer product is also seen in the cooler *Profile 3* instrument (Eq. 11a), which emphasises that the lower flow tube temperature encourages a softer route to ionisation.

In the previous work, Španěl et al., (1997)[21] reported the production of only two product ions. These were the charge transfer product ($C_4H_6O_2^{+\bullet}$) at 65% which compares to 81% within this study; and the fragmentation product ($C_2H_3O^+$) at 35% (which compares to 1% in this study). Replacing He with N_2 within the *Profile 3* causes the charge transfer product to increase and the fragmentation product ion to decrease, substantially. This is due to the heavier relative molecular mass of N_2 compared to He which absorbs a greater quantity of the kinetic energy of the $O_2^{+\bullet}$ radicals, reducing the energy of these species, subsequently allowing for a softer pathway to ionisation of ethanol. This does however mean that the branching ratios need to be updated for this molecule within the library.

2.2.4 Acetaldehyde

A substantial difference is also seen within acetaldehyde, regarding the branching ratios between the two instruments. In the same manner as the previously discussed reactions, the adduct formation product ($C_2H_4O.NO^+$, Eq. 12b) exhibits a much lower branching ratio within the Voice200(*Infinity*) instrument, compared to the *Profile 3*. This is down to the increased flow tube temperature which inhibits the dative bonding of the NO^+ reagent ion with the analyte species. In accordance with this, the increased temperature induces the hydrogen atom bond fission and causes a higher proportion of the H-abstraction product within the Voice200(*Infinity*) instrument (Eq. 12a).

$$NO^+ + C_2H_4O \rightarrow C_2H_3O^+ + HNO$$ (*m/z* 43) [*81*, **96**] (Eq. 12a)

$$\rightarrow C_2H_4O.NO^+$$ (*m/z* 74) [*19*, **4**] (Eq. 12b)

Previously, the reaction of NO^+ with acetaldehyde was reported to only produce the H-abstraction product at 100 % branching (within the *Profile 3* using He as the carrier gas[21]). By switching the carrier gas to N_2, this has enabled for a substantial production of the adduct ion ($C_2H_4O.NO^+$). N_2 quenches some of the energy from the NO^+ reagent ions within the flow tube and inherently this lowers the impact energy of NO^+ with acetaldehyde, allowing for the formation of the adduct. The use if N_2 is so effective in producing the adduct in this reaction, that even the Voice200(*Infinity*) at the much higher temperature of the flow tube (and extra electric field the end of the flow tube), is able to detect it.

2.2.5 Acetic Acid

Acetic acid demonstrated the adduct formation product ($CH_3COOH.NO^+$, Eq. 13a) to be the major product ion within both instruments, although the Voice200(*Infinity*) instrument also demonstrated the production of the fragmentation product (CH_3O^+, Eq. 13b). As with some of the other analytes, it was

initially uncertain as to whether the fragmentation product at *m/z* 43 was real or not. Analysing the blank analysis as well as the time series of the experiment showed that *m/z* 43 increased significantly in the Voice200(*Infinity*) instrument, as headspace of acetic acid was added to the glass vessel.

$NO^+ + CH_3COOH \rightarrow CH_3COOH.NO^+$ (*m/z* 90) [*100*, **88**] (Eq. 13a)

$\rightarrow C_2H_3O^+ + HNO_2$ (*m/z* 43) [*0*, **12**] (Eq. 13b)

The reaction rate for these compounds with NO^+ were however slow, being 0.9×10^{-9} cm^3 s^{-1} for the *Profile 3* and 0.2×10^{-9} cm^3 s^{-1} for the Voice200(*Infinity*), as shown in Table 2. Despite this, it is necessary for the kinetics library and the literature to be updated, regarding the branching ratios of the reaction of acetic acid with NO^+.

The previous work (in which He was used within the *Profile 3*)[24] shows the production of only the adduct, which agrees with the results from this study when using N_2 as the carrier gas. It should be highlighted however that the flow tube conditions of the Voice200(*Infinity*) induces the formation of the fragment ion ($C_2H_3O^+$) and therefore the branching ratio values from the previous study of NO^+ with acetic acid[24] should not be directly incorporated into the Voice200(*Infinity*) library.

2.2.6 Acetone

The major product ion exhibited by both instruments was the adduct formation species ($C_3H_6O.NO^+$, Eq. 14a). In the *Profile 3* this was determined to be the only species produced, whereas in the Voice200(*Infinity*) the increased flow tube temperature encouraged the formation of the fragmentation product (CH_3O^+, Eq. 14b) as well as the product ion radical species, $C_3H_6O^{+\bullet}$ (Eq. 14c).

$NO^+ + C_3H_6O \rightarrow C_3H_6O.NO^+$ (*m/z* 88) [*100*, **81**] (Eq. 14a)

$\rightarrow C_2H_3O^+ + CH_3NO$ (*m/z* 43) [*0*, **12**] (Eq. 14b)

$\rightarrow C_3H_6O^{+\bullet} + NO^\bullet$ (*m/z* 58) [*0*, **7**] (Eq. 14c)

The ability for the Voice200(*Infinity*) instrument to be able to form the fragmentation and charge transfer ion radical species in Eq. 14b and 14c, requires the high thermal energy input from the Voice200(*Infinity*) flow tube. Likewise, to the previous analytes discussed, *m/z* 43 was deemed to be a real product as the signal increased significantly and proportionally, when acetone headspace was added to the glass vessel. In agreement with this work, the previous branching ratio determined using the *Profile 3* with a He carrier gas also produced 100 % of the $C_3H_6O.NO^+$ adduct[21].

2.2.7 Ethyl acetate

A significant number of minor ions were detected from the reaction of ethyl acetate with NO^+ within the Voice200(*Infinity*), although only the major adduct ion ($C_4H_8O_2.NO^+$, Eq. 15a) was seen within *Profile 3* instrument. Like the other analytes, the higher flow tube temperature of the Voice200(*Infinity*) reduces the branching route of adduct formation within this instrument. Interestingly, ethyl acetate produces more minor product ions within the Voice200(*Infinity*) compared to the other species, which is down to the favourable energetics of these branching pathways.

$NO^+ + C_4H_8O_2 \rightarrow C_4H_8O_2.NO^+$ (*m/z* 118) [*100*, **85**] (Eq. 15a)

$\rightarrow C_2H_3O^+ + C_2H_5NO_2$ (*m/z* 43) [*0*, **7**] (Eq. 15b)

$\rightarrow C_2H_5O_2^+ + C_2H_3NO$ (*m/z* 61) [*0*, **6**] (Eq. 15c)

$\rightarrow C_4H_8O_2^{+\bullet} + NO^{\bullet}$ (*m/z* 88) [*0*, **1**] (Eq. 15d)

$\rightarrow C_4H_7O_2^{+} + HNO$ (*m/z* 87) [*0*, **1**] (Eq. 15e)

Although $C_4H_8O_2^+$ (Eq. 15d) and $C_4H_7O_2^+$ (Eq. 15e) were observed to only produce a branching pathway of 1%, these product ions were seen to correlate linearly with the major product ion ($C_4H_8O_2.NO^+$); and their class of reactions (charge transfer and H-abstraction for Eq. 15d and 15e, respectively) are entirely plausible. The presence of $C_2H_3O^+$ (*m/z* 43) and $C_2H_5O_2^+$ (*m/z* 61) were also confirmed to be real products by their strong correlation with the production of the major adduct ion (Eq. 15a), as well as their increase in the time series of the MIM experiment, on the addition of ethyl acetate headspace (as well as absence in the blank analysis).

A higher percentage of the $C_4H_8O_2.NO^+$ (100%) was recorded in this work (*Profile 3*) when using N_2 as the carrier gas, as opposed to the work of Španěl et al., (1998)[24] who reported 90% of this product ion, along with 10% of the fragmentation product ($C_2H_3O^+$) when He was used. The larger mass of the N_2 absorbs a greater fraction of the kinetic energy exhibited by the NO^+ reagent ions and allows for a slower collisional speed to occur between the reagent ions and the analyte molecules. This causes softer collisions which is why only the adduct is seen when using N_2 in the *Profile 3*, as in this work.

2.2.8 Ethanol

The major reaction pathway of ethanol with NO^+ results in the formation of the H-abstraction product ($C_2H_5O^+$, Eq. 16a). There is however a minor proportion of the adduct ($C_2H_6O.NO^+$, Eq. 16b) which is formed in the *Profile 3* instrument, due to the lower flow tube temperature exhibited.

$NO^+ + C_2H_6O \rightarrow C_2H_5O^+ + HNO$ (*m/z* 45) [*96*, **100**] (Eq. 16a)

$\rightarrow C_2H_6O.NO^+$ (*m/z* 76) [*4*, **0**] (Eq. 16b)

The previous branching ratios for this reaction in He within the *Profile 3*[23] demonstrated only a single pathway, which was the H-abstraction mechanism (Eq. 16a). The use of N_2 induces a softer method of ionisation compared to He, as discussed for the previous molecules. This is why the addition of the adduct product ($C_2H_6O.NO^+$) is observed within N_2, although small. The Voice200(*Infinity*) also gives 100% of the H-abstraction pathway, although the inclusion of the high temperature flow tube counteracts the lower collisional energy of the NO^+ ions.

2.3 O_2^+ Reactions

The O_2^+ reagent ions are less frequently used for the analysis of gaseous species (compared to H_3O^+ or NO^+), due to the highly energetic nature of this radical ion, often causing extensive fragmentation to organic species. Nonetheless, O_2^+ may be used to measure some key organic species, such as isoprene[14]. To gain the holistic understanding of the differences occurring within the ion-molecule reactions across the positive ions between the *Profile 3* and Voice200(*Infinity*), the comparison of the O_2^+ reactions are discussed in this section. It was found that significant differences in branching ratios occurred across all analyte species. This essential finding therefore shows that for the O_2^+ reactions, the branching ratios must be updated for analyte species within the kinetics library to allow for the accurate concentration calculation of VOCs using the Voice200(*Infinity*).

2.3.1 1-Propanol

Five ionic products were detected from the reaction of O_2^+ with 1-propanol. These included $C_3H_8O^{+\bullet}$ produced by the proton transfer reaction (Eq. 17a); $C_3H_7O^+$ produced by the H-abstraction reaction (Eq. 17c); as well as CH_3O^+ (Eq. 17b), $CH_5O_2^+$ (Eq. 17d) and $C_2H_2O^{+\bullet}$ (Eq. 17e) produced as a result of fragmentation.

$O_2^{+\bullet} + C_3H_8O \rightarrow C_3H_8O^{+\bullet} + O_2$ (*m/z* 60) [*10*, **3**] (Eq. 17a)

$\rightarrow CH_3O^+ + C_2H_5^{\bullet} + O_2$ (*m/z* 31) [*76*, **48**] (Eq. 17b)

$\rightarrow C_3H_7O^+ + HO_2^{\bullet}$ (*m/z* 59) [*2*, **24**] (Eq. 17c)

$\rightarrow CH_5O_2^+ + C_2H_3O^{\bullet}$ (*m/z* 49) [*6*, **12**] (Eq. 17d)

$\rightarrow C_3H_6^{+\bullet} + H_2O + O_2$ (*m/z* 42) [*2*, **10**] (Eq. 17e)

Unsurprisingly, the soft proton transfer pathway (Eq. 17a) has a larger distribution within the *Profile 3* compare to the Voice200(*Infinity*), which is down to the lower flow tube temperature. There is also a much more even distribution across the pathways within the Voice200(*Infinity*) compared to the *Profile 3*. This demonstrates the harsher conditions of the increased flow tube temperature as well as the presence of a known electric field at the end of the Voice200(*Infinity*), which not present in the *Profile 3* and causes an increase in the speed of ions as they collide in the flow tube. This therefore explains the higher proportion of the other ions ($C_3H_7O^+$, $CH_5O_2^+$, and $C_2H_2O^{+\bullet}$) in the Voice200(*Infinity*), which take only a minor fraction of the branching ratios within the *Profile 3*.

The previous work by Španěl et al., (1997)[23] reported a 90 % production of the CH_3O^+ fragment, as well as a 10% production of $C_3H_6^+$ at *m/z* 42. The CH_3O^+ branching ratio differs significantly in our work from the literature, although a branching ratio of 10% of $C_3H_6^+$ is also seen in the Voice200(*Infinity*) results.

The lower proportion of CH_3O^+ within the *Profile 3* using N_2 (compared to He) may be partially explained by the replacement of the soft ionisation charge transfer product ($C_3H_8O^{+\bullet}$) which is caused by the reduced collisional energy of the $O_2^{+\bullet}$ species (as discussed). Furthermore, what are minor ions within the *Profile 3* (in both He and N_2) are major ions within the Voice200(*Infinity*). As a result, the branching ratio values acquired by the *Profile 3* using He should not be directly overlaid with the use if the Voice200(*Infinity*), using N_2.

2.3.2 2-Propanol

A significant difference in branching ratio is seen between the two instruments for the major ions $C_2H_5O^+$ (Eq. 18a) and $C_3H_7O^+$ (Eq. 18b). Similar branching ratios were however seen between the instruments for the minor branching pathways, producing $C_2H_4O^{+\bullet}$ (Eq. 18c) and $C_3H_7^+$ (Eq. 18d).

$O_2^{+\bullet} + C_3H_8O \rightarrow C_2H_5O^+ + CH_3O_2^{\bullet}$ (*m/z* 45) [*60*, **77**] (Eq. 18a)

$\rightarrow C_3H_7O^+ + HO_2^{\bullet}$ (*m/z* 59) [*22*, **4**] (Eq. 18b)

$\rightarrow C_2H_4O^{+\bullet} + CH_4 + O_2$ (*m/z* 44) [*4*, **3**] (Eq. 18c)

$\rightarrow C_3H_7^+ + HO^{\bullet} + O_2$ (*m/z* 43) [*14*, **16**] (Eq. 18d)

The branching ratios within the current library are taken from Španěl et al., (1997)[23] and report a 100% reaction pathway of the production of the charge transfer product ($C_2H_5O^+$, Eq. 18a). Although this product is also the most dominant in both instruments when using N_2, there are a substantial fraction of other products ($C_3H_7O^+$, $C_2H_4O^{+\bullet}$, $C_3H_7^+$) which also need to be accounted for when calculating the absolute concentration of species, from the Voice200(*Infinity*) instrument.

2.3.3 2,3-Butanedione

A surprisingly simple set of branching pathways was determined for the reaction between O_2^+ and 2,3-butanedione, in which only two pathways were concluded. These were the fragmentation pathway, producing the $C_2H_3O^+$ ion (Eq. 19b) as well as the charge transfer product $C_4H_6O_2^{+\bullet}$ (Eq. 19a). Unsurprisingly, the fragmentation pathway is significantly more dominant in the Voice200(*Infinity*) compared to the *Profile 3*, due to the increased speed of the ions as well as the higher flow tube

temperature causing a higher fraction of collisions to overcome the activation energy barrier for Eq. 19b.

$$O_2^{+\bullet} + C_4H_6O_2 \rightarrow C_4H_6O_2^{+\bullet} + O_2\ (m/z\ 86)\ [\mathit{33}, \mathbf{5}]\ \text{(Eq. 19a)}$$

$$\rightarrow C_2H_3O^+ + C_2H_5^{\bullet} + O_2\ (m/z\ 43)\ [\mathit{67}, \mathbf{95}]\ \text{(Eq. 19b)}$$

A higher proportion of the fragment ion was detected in the *Profile 3* when using He in the work of Španěl et al., (1997)[21]. Inevitably, the use of N_2 also increased the proportion of the soft ionisation charge transfer product ($C_4H_6O_2^{+\bullet}$) which is due to the elasticity of collisions of the O_2^+ ions with N_2 reducing the relative energy of the O_2^+ reagent ions, as previously discussed. This allows for the softer ionisation pathway (Eq. 19a) to increase in its branching ratio, when using N_2 as the carrier gas.

2.3.4 Acetaldehyde

H-abstraction was the major branching pathway in both instruments, although the Voice200(*Infinity*) exhibited a slightly higher fraction of the major ion ($C_2H_3O^+$, Eq. 20b), compared to the *Profile 3*. This is because of the higher energy associated with the O_2^+ ions within the Voice200(*Infinity*) from the increased flow tube temperature and the extra electric field found at the end of the flow tube. Although the difference here is minor between the instruments, the kinetic library associated with the Voice200(*Infinity*) should still be updated.

$$O_2^+ + C_2H_4O \rightarrow C_2H_4O^{+\bullet} + O_2\ (m/z\ 44)\ [\mathit{7}, \mathbf{3}]\ \text{(Eq. 20a)}$$

$$\rightarrow C_2H_3O^+ + HO_2^{\bullet}\ (m/z\ 43)\ [\mathit{93}, \mathbf{97}]\ \text{(Eq. 20b)}$$

The results from this study were considerably different to those previously reported in the literature. Španěl et al., (1997)[21] report an almost even split in branching pathways between $C_2H_4O^{+\bullet}$ (55%) and $C_2H_3O^+$ (45%). It is surprising that for this reaction in particular, the branching ratio of the charge transfer product is significantly lower, in the presence of a N_2 carrier gas and deviates away from the general pattern observed within the other analyte species investigated in this study.

2.3.5 Acetic Acid

For the reaction between acetic acid and $O_2^{+\bullet}$, the major products were the charge transfer ion ($CH_3COOH^{+\bullet}$, Eq. 21a) as well as the fragment ion (CH_3O^+, Eq. 21b). A larger branching ratio of the soft charge transfer pathway was exhibited by the *Profile 3* due to the lower flow tube temperature conditions, whereas a larger fraction of the fragment ion (CH_3O^+, Eq. 21b) was exhibited by the Voice200(*Infinity*), due to the increased temperature. A small fraction of the minor ion $COOH^+$ (eq. 21c) was also shown by the Voice200(*Infinity*) due to an excess of collisional energy acquired by the $O_2^{+\bullet}$, within the Voice200(*Infinity*) flow tube.

$$O_2^{+\bullet} + CH_3COOH \rightarrow CH_3COOH^{+\bullet} + O_2\ (m/z\ 60)\ [\mathit{66}, \mathbf{58}]\ \text{(Eq. 21a)}$$

$$\rightarrow C_2H_3O^+ + HO^{\bullet} + O_2\ (m/z\ 43)\ [\mathit{34}, \mathbf{40}]\ \text{(Eq. 21b)}$$

$$\rightarrow COOH^+ + CH_3O_2^{\bullet}\ (m/z\ 45)\ [\mathit{0}, \mathbf{2}]\ \text{(Eq. 21c)}$$

Analogous to acetaldehyde, these branching ratios are similar although it is still necessary to use the updated branching ratio values within the Voice200(*Infinity*) instrument, compared to the *Profile 3* values from the previous literature[24].

The previous work in He with the *Profile 3* showed an even split between the two product ions $CH_3COOH^{+\bullet}$ and CH_3O^+[24]. As would be expected based on the pattern established within this study, the

proportion of the charge transfer product increases and the branching ratio of the fragmentation product decreases in the presence of a N_2 carrier gas due to the elasticity of collisions changing between the reagent ion and the carrier gas.

2.3.6 Acetone

Two major product ions were detected by both instruments, which were the charge transfer product ($C_3H_6O^{+\bullet}$, Eq. 22a), as well as the fragmentation ion ($C_2H_3O^+$, Eq. 22b). The ratios however invert, in which the *Profile 3* instrument majorly produces the charge transfer product, whereas the Voice200(*Infinity*) instrument majorly produces the fragmentation product. This is down to the higher temperature and extra electric field found at the end of the Voice200(*Infinity*) flow tube.

$O_2^{+\bullet} + C_3H_6O \rightarrow C_3H_6O^{+\bullet} + O_2$ (*m/z* 58) [*65*, **34**] (Eq. 22a)

$\rightarrow C_2H_3O^+ + CH_3O_2$ (*m/z* 43) [*35*, **65**] (Eq. 22b)

$\rightarrow C_2H_2O^+ + CH_4 + O_2$ (*m/z* 42) [*0*, **1**] (Eq. 22c)

There is a small change in branching ratio here, compared to the previous work by Španěl et al., (1997)[24]. Authors reported a branching ratio of 60% for $C_3H_6O^{+\bullet}$ and 40% for $C_2H_3O^+$ (He), which compares with 65% and 35 % in this work (N_2), respectively. The branching ratio of the charge transfer product increases by 5% in N_2 (when comparing the *Profile 3* instruments) which conforms to the pattern of softer ionisation pathways occurring within a N_2 carrier gas. Nonetheless, a significantly larger difference in branching ratios is seen between the initial work using He and the Voice200(*Infinity*) instruments (now routinely used); and therefore, these branching ratios need to be updated within the kinetic library of the Voice200(*Infinity*).

2.3.7 Ethyl acetate

Ethyl acetate exhibited multiple ionic products in both instruments. The major pathways were however evenly distributed across the production of the fragmentation products $C_2H_5O_2^+$ (Eq. 23a), $C_2H_5O^+$ (Eq. 23b) and $C_2H_3O^+$ (Eq. 23c). There was however also a small amount of the charge transfer product ($C_4H_8O_2^{+\bullet}$, Eq. 23d) also produced. In agreement with the previously discussed analytes, the soft charge transfer product (Eq. 23d) branching ratio is significantly higher in the *Profile 3* compared to the Voice200(*Infinity*).

$O_2^{+\bullet} + C_4H_8O_2 \rightarrow C_2H_5O_2^+ + C_2H_3O_2^\bullet$ (*m/z* 61) [*24*, **38**] (Eq. 23a)

$\rightarrow C_2H_5O^+ + C_2H_3O_3^\bullet$ (*m/z* 45) [*24*, **19**] (Eq. 23b)

$\rightarrow C_2H_3O^+ + C_2H_5O_3^\bullet$ (or $C_2H_5O^\bullet + O_2$) (*m/z* 43) [*25*, **33**] (Eq. 23c)

$\rightarrow C_4H_8O_2^{+\bullet} + O_2$ (*m/z* 88) [*12*, **6**] (Eq. 23d)

$\rightarrow C_3H_5O_2^+ + CH_3O_2^\bullet$ (*m/z* 73) [*12*, **1**] (Eq. 23e)

$\rightarrow C_4H_7O_2^+ + HO_2^\bullet$ (*m/z* 87) [*3*, **1**] (Eq. 23f)

$\rightarrow C_4H_8O_2.O_2^{+\bullet}$ (*m/z* 120) [*0*, **1**] (Eq. 23g)

$\rightarrow C_2H_2O^+ + C_2H_6 + O_3$ (*m/z* 42) [*0*, **1**] (Eq. 23h)

It was initially questionable as to whether $C_2H_5O_2^+$ (Eq. 23a) at *m/z* 61 was a real product or not. This is because the hydrate of the common *m/z* 43 product ion ($C_2H_3O^+$) would also equate to an *m/z* value of 61. Based on the previous literature however[24], as well as the thermodynamics of this process and

the relative branching ratio of $C_2H_3O^+$ at *m/z* 43, it was determined that the signal at *m/z* 61 equated to a real ionic product ($C_2H_5O_2^+$).

The work of Španěl et al., (1998)[24] reported multiple products of $C_2H_5O_2^+$ (40%), $C_2H_3O^+$ (20%), $C_2H_5O^+$ (20%) and CH_3O^+ (20%) when using the *Profile 3* with a He carrier gas. The Voice200(*Infinity*) with N_2 showed very close branching ratios of 38% for $C_2H_5O_2^+$ and 19% for $C_2H_5O^+$.

The branching ratio of $C_2H_3O^+$ in our study was however much larger (33%) compared to that of Španěl et al., (1998)[24]. This is most likely down to the increased thermal energy within the flow tube causing a greater proportion of fragmentation to occur in this instrument. This is reflected in the additional minor ions produced by the Voice200(*Infinity*) which were not reported in the previous work. This may however be down to the increased sensitivity of the Voice200(*Infinity*) compared to the *Profile 3*. Another significant difference is that the *Profile 3* using He produced CH_3O^+ (*m/z* 31) at 20% branching, which was not detected at all in an N_2 carrier gas, in either instrument.

2.3.8 Ethanol

Only two major products were identified from the reaction of ethanol with O_2^+. These were the H-abstraction product ($C_2H_5O^+$, Eq. 24a) as well as the fragmentation product (CH_3O^+, Eq. 24b). A minor branching pathway was also seen in the *Profile 3* for the charge transfer product (Eq. 24c), which was not detected in the Voice200(*Infinity*).

$$O_2^{+\bullet} + C_2H_6O \rightarrow C_2H_5O^+ + HO_2^\bullet \ (m/z\ 45)\ [\mathit{22}, \mathbf{65}]\ \text{(Eq. 24a)}$$

$$\rightarrow CH_3O^+ + CH_3O_2^\bullet \ (m/z\ 31)\ [\mathit{73}, \mathbf{35}]\ \text{(Eq. 24b)}$$

$$\rightarrow C_2H_6O^{+\bullet} + O_2 \ (m/z\ 46)\ [\mathit{5}, \mathbf{0}]\ \text{(Eq. 24c)}$$

To compare with the literature which was used to form the library for the Voice200(*Infinity*) instrument, Španěl et al., (1997)[23] report the production of only two ionic products. These are $C_2H_5O^+$ which has a 75% branching ratio in the *Profile 3* with He (compared to 22% in *Profile 3*, N_2); and $C_2H_6O^{+\bullet}$ which has a 25% branching ratio in the *Profile 3* with He (compared to 5% in *Profile 3*, N_2). The major ion for the *Profile 3* (N_2 work) was the fragment species CH_3O^+ (73%, Eq. 24b) which was not reported in Španěl et al., (1997)[23] when using a He carrier gas. There are therefore huge differences in the branching ratios between the former *Profile 3* He work[23] and this work. The former branching ratios should not be used in the library of the Voice200(*Infinity*).

2.4 *R*-Limonene Reactions

Much more fragmentation was observed in the reactions of *R*-limonene with the three separate reagent ions in both instruments within N_2. This is different to what was previously thought within the literature. Although the major ions are similar to the previously literature[31], the use of the Voice200 (as opposed to the *Profile 3*) has highlighted the presence of many major minor ions within all three ion-molecule reactions which need to be addressed and are discussed in this section.

The major ions for this comparison are found in Table 1, although a significant fraction for all three reactions gave an 'Other' fragment which is the accumulation of many fragmentation products. To demonstrate the extent of fragmentation exhibited by these different products within the *Profile 3* and Voice200(*Infinity*) using an N_2 carrier gas, the comparison fragmentation spectra for *R*-limonene with H_3O^+, NO^+ and O_2^+ in both instruments are shown in Fig. 1. The major product ions are reported in table 1 and the minor products reported in this section are highlighted in red in each spectrum. Although the following minor ions are all very negligible, they cumulatively are an important fraction of the branching ratio.

For H_3O^+, the minor ions are possibly $C_5H_{15}^+$ (5%), $C_7H_{11}^+$ (2%), $C_5H_9^+$ (2%), $C_4H_{11}^+$ (6%) and $C_{10}H_{15}^+$ (5%) in the *Profile 3*. This compares to the Voice200(*Infinity*) for which we contemplate the minor ions

to be $C_7H_{11}^+$ (5%), $C_7H_9^+$ (3%), $C_5H_9^+$ (1%), $C_5H_{15}^+$ (4%), $C_6H_{13}^+$ (1%), $C_8H_{11}^+$ (1%), $C_4H_{11}^+$ (7%), $C_5H_7^+$ (1%).

For NO^+ in the *Profile 3*, a signal at *m/z* 104 was a minor ion with the possible formula of $C_8H_8^+$ (2%) as well as $C_4H_{10}^+$ (5%) at *m/z* 88. For the Voice200(*Infinity*), $C_2H_3O^+$ (4%), $C_2H_5O^+$ (3%), $C_4H_9^+$ (1%), $C_5H_{12}^+$ (2%), $C_6H_8^+$ (1%), $C_4H_{10}NO^+$ (2%), $C_7H_8^+$ (4%), $C_7H_9^+$ (3%), $C_7H_{10}^+$ (2%), $C_7H_{11}^+$ (1%), $C_8H_8^+$ (2%), $C_8H_{11}^+$ (1%), $C_8H_{12}^+$ (1%), $C_8H_{13}^+$ (1%), and $C_9H_{13}^+$ (3%).

For O_2^+, the *Profile 3* exhibited the minor ions $C_5H_8^+$ (4%), $C_6H_8^+$ (3%), $C_6H_9^+$ (2%), $C_7H_8^+$ (6%), $C_7H_{11}^+$ (4%), $C_8H_{11}^+$ (6%), $C_8H_{12}^+$ (12%). The minor ions in the Voice200(*Infinity*) were seen to be $C_2H_3O^+$ (8%), $C_2H_5O^+$ (1%), $C_5H_8^+$ (9%), $C_6H_7^+$ (1%), $C_6H_8^+$(4%), $C_6H_9^+$ (2%), $C_6H_{10}^+$ (1%), $C_7H_8^+$ (6%), $C_7H_{11}^+$ (3%), $C_8H_{11}^+$ (8%) and $C_8H_{12}^+$ (5%).

Author Information

Corresponding Author

Patrik Španěl - *J. Heyrovsky Institute of Physical Chemistry, 3, Dolejškova 2155, Libeň, 182 00 Praha 8, Czech Republic*

Authors

Stefan J Swift - *J. Heyrovsky Institute of Physical Chemistry, 3, Dolejškova 2155, Libeň, 182 00 Praha 8, Czech Republic*
Nicola Sixtová - *J. Heyrovsky Institute of Physical Chemistry, 3, Dolejškova 2155, Libeň, 182 00 Praha 8, Czech Republic*
Nicholas Demarais - *Syft Technologies, 68 Saint Asaph Street, Christchurch Central City, Christchurch 8011, New Zealand*
